# Tissue Distribution of the *Ehrlichia muris*-Like Agent in a Tick Vector

**DOI:** 10.1371/journal.pone.0122007

**Published:** 2015-03-17

**Authors:** Geoffrey E. Lynn, Jonathan D. Oliver, Curtis M. Nelson, Roderick F. Felsheim, Timothy J. Kurtti, Ulrike G. Munderloh

**Affiliations:** Department of Entomology, University of Minnesota, 219 Hodson Hall, 1980 Folwell Ave., St. Paul, MN 55108, United States of America; Kansas State University, UNITED STATES

## Abstract

Human pathogens transmitted by ticks undergo complex life cycles alternating between the arthropod vector and a mammalian host. While the latter has been investigated to a greater extent, examination of the biological interactions between microbes and the ticks that carry them presents an equally important opportunity for disruption of the disease cycle. In this study, we used *in situ* hybridization to demonstrate infection by the *Ehrlichia muris*-like organism, a newly recognized human pathogen, of *Ixodes scapularis* ticks, a primary vector for several important human disease agents. This allowed us to assess whole sectioned ticks for the patterns of tissue invasion, and demonstrate generalized dissemination of ehrlichiae in a variety of cell types and organs within ticks infected naturally via blood feeding. Electron microscopy was used to confirm these results. Here we describe a strong ehrlichial affinity for epithelial cells, neuronal cells of the synganglion, salivary glands, and male accessory glands.

## Introduction

The incidence of tick-borne zoonoses has been increasing worldwide, primarily in moderate climate zones, which is attributable in part to the expanded geographical distribution and abundance of vector ticks. Ongoing changes in climate and habitat, as well as human land usage frequently alter the composition of local flora and fauna in favor of ticks and their hosts [1–10]. While some of the causative agents have been known for decades, others have only recently been identified as human pathogens. One such organism, a species closely related to *Ehrlichia muris*, was cultured from a human patient diagnosed with ehrlichiosis acquired in Wisconsin in 2009 [11]. Previously undescribed in that area, as of 2013, 67 cases of illness caused by the *E*. *muris-*like agent (EML) have been documented with probable exposure in Minnesota and Wisconsin (2009–2013)[12]. Clinical presentation is similar to human anaplasmosis and human monocytic ehrlichiosis, with symptoms including fever, headache, myalgia, malaise, and nausea [11,12]. EML organisms have been identified via polymerase chain reaction (PCR) in blacklegged ticks (*Ixodes scapularis*) [11,13] and isolated from an engorged adult *I*. *scapularis* [14], suggesting this species as the probable primary vector to humans.

In order to identify possible points of intervention related to prevention and treatment of parasitic diseases, it is invaluable to understand the development and biological requirements of the etiologic agent and how it interacts with each of its hosts [15]. Several medically important species within the family *Anaplasmataceae*, to which EML belongs, have been well-studied in vertebrate hosts, including the zoonotic pathogen *Anaplasma phagocytophilum*, and the veterinary pathogen *Anaplasma marginale*, which causes bovine anaplasmosis. Within the genus *Ehrlichia*, the species most frequently responsible for human illness, *Ehrlichia chaffeensis*, has been studied extensively *in vitro* and in human tissues [16–18], and EML infection in mice has been evaluated as a potential model of human ehrlichiosis [19]. Comparatively, understanding of the biological interaction between *Anaplasmataceae* and their tick vectors lags behind that of mammalian hosts, especially those with medical significance. In particular, description of specific sites of infection within ticks has relied largely upon detection of pathogen DNA in tick tissues using the polymerase chain reaction (PCR). A number of factors contribute to the difficulty of visually identifying these bacteria in ticks, including their intracellular niche, small size, polymorphic shape, low pathogen load of some species, as well as the autofluorescence of digestive byproducts and certain anatomical features of ticks. It is important to surmount these obstacles in order to improve our understanding of individual pathogens, their unique characteristics such as cell/tissue tropisms and processes of acquisition and transmission, and to evaluate the contrasts between their dual life histories in mammalian hosts and tick vectors.

Among the *Anaplasmataceae*, only the life cycle of *A*. *marginale* has been described extensively *in vivo* in ticks. Using light and electron microscopy, morphologic features unique to *in vivo* infection have been imaged, and various bacterial life stages identified in a wide range of tissues in *Dermacentor andersoni*, with the salivary glands and gut muscle cells shown to be important sites for replication and transmission, respectively [15,20–23]. In contrast, among *Anaplasmataceae* commonly infecting humans, published literature on tissue localization is limited to *A*. *phagocytophilum*. This includes identification in salivary glands, gut, and hemolymph using PCR, staining, and light microscopy [24–26] as well as a single electron microscopy study showing morulae (membrane bound bacterial inclusions within the cytoplasm of host cells) in tick gut muscle cells, salivary glands, and ganglia [27]. *Ehrlichia chaffeensis* has yet to be imaged in ticks, while the ultrastructural features of *E*. *canis* and *E*. *ruminantium* have been shown in several organs of experimentally infected ticks [28,29]. In addition, there is a single publication containing micrographs of ehrlichial organisms in the salivary glands of wild caught ticks [30].

To address this deficit in knowledge and determine potential targets for disrupting pathogen acquisition and transmission, we undertook an in-depth visual investigation of EML infection in nymphal, male, and female *I*. *scapularis* using *in situ* hybridization (ISH) and electron microscopy.

## Materials and Methods

### Tick infection

Larval *I*. *scapularis* were hatched from gravid females obtained from Oklahoma State University and allowed to feed to repletion on 10-week old outbred Syrian hamsters (*Mesocricetus auratus*) inoculated intraperitoneally with ISE6 cell culture [31,32] infected with the EmCRT (*Ehrlichia muris*-like agent Camp Ripley Tick) isolate. Larvae were assumed pathogen free given that colony-reared adults are fed on pathogen-free animal hosts and ehrlichiae have not been shown to be transovarially transmitted. The EmCRT isolate was cultured from an engorged female *I*. *scapularis* collected at Camp Ripley, Minnesota in the fall of 2011. Following euthanasia, PCR was performed on blood drawn from a single hamster via cardiac puncture eight days post inoculation (d.p.i.) to confirm EML infection. The remaining hamsters were then infested with larvae the following day (9 d.p.i.). Blood was collected as described previously from hamsters for PCR once most larvae had fed to repletion (15 d.p.i.) to confirm tick exposure to EML.

Animal research was carried out in accordance with the recommendations in the Guide for the Care and Use of Laboratory Animals of the National Institutes of Health [33]. Sodium pentobarbital was used to sedate rodents for tick application, and carbon dioxide overexposure was used for euthanization of hamsters, following a protocol approved by The Institutional Animal Care and Use Committee (IACUC) of the University of Minnesota (# 1307–30753A). Fully-engorged larvae were surface cleaned with sodium hypochlorite diluted to 0.01% in water and rinsed with Milli-Q (Millipore Corporation, Billerica, MA, USA) filtered water. Cleaned ticks were housed in 5 mL vented polystyrene tubes (BD Biosciences, Canaan, CT, USA) and stored in a glass desiccator over a saturated solution of K_2_SO_4_ (∼97% humidity) at room temperature (20–22 degrees Celcius) under a 16 hour light, 8 hour dark photoperiod [34]). Following molting, five nymphs were assessed for infection using PCR, and the remainder were fed on naïve hamsters 8–12 weeks later and allowed to molt into adults. Adults were then tested for ehrlichial infection by PCR.

### Determination of tick infection by PCR

Ticks used for PCR were washed as described above, placed individually in sterile 1.5 ml micro-centrifuge tubes and sliced into 2–3 pieces using sterile 25G x 5/8 inch needles (BD, Franklin, NJ, USA) and fine forceps. To avoid carry-over of DNA, fresh needles and forceps were used for individual ticks. Pieces were then submerged in 300 μl Cell Lysis Solution (Puregene Core Kit A; Qiagen Sciences, Valencia, CA, USA), vortexed, heated to 75°C for 5 min and allowed to further dissolve overnight at room temperature. DNA was extracted from ticks using the Puregene Core Kit A per manufacturer’s instructions (Qiagen) and PCR was performed using PER 1 (5'TTTATCGCTATTAGATGAGCCTATG3') and PER 2 (5'CTCTACACTAGGAATTCCGCTAT3') primers [35] with GoTaq DNA polymerase (Promega, Madison, WI, USA) to amplify ehrlichial DNA. These primers target the 16S rDNA and produce a 451 bp product that was visualized after agarose gel electrophoresis and GelGreen staining (Biotium, Hayward, CA, USA) to verify successful PCR-amplification. The primer set can be used to identify members of the genera *Anaplasma* and *Ehrlichia* (including EML), but not does not amplify sequence of the *I*. *scapularis* symbiont, *Rickettsia buchneri* [36].

### In Situ Hybridization (ISH)

Sections of the dorsal carapace were removed from surface-sterilized male, female, and nymphal *I*. *scapularis*, and ticks were fixed overnight in a 10% buffered formalin solution (Fisher Scientific, Hampton, NH, USA) at 4°C. Fixed specimens were washed in 70% ethanol and positioned in groups of four in 1.2% agarose gel to retain planar position. Blocks of gel were then submitted to the Masonic Cancer Center Comparative Pathology Laboratory at the University of Minnesota where they were paraffin embedded, sectioned at 4 μm thickness, and placed onto Superfrost Plus microscope slides (Fisher Scientific, Minneapolis, MN, USA).

Slides were baked at 60°C for 60 min prior to paraffin removal with xylenes (Fisher Scientific). Next, ISH was performed using the QuantiGene ViewRNA ISH Tissue 1-Plex Assay for RNA (Affymetrix, Santa Clara, CA, USA) with Gill’s hematoxylin and DAPI (4',6-diamidino-2-phenylindole) used as described in the manufacturer’s protocol to provide tissue- and cellular context. An EmCRT-specific DNA probe from Affymetrix containing a set of 20 oligonucleotide pairs targeting the EmCRT 23S rRNA (GenBank accession ID# BankIt1793496 E_muris_ KP702294) was hybridized to the bacterial RNA. The sequence selected to construct the probe was obtained using the EmCRT Illumina sequencing trace file downloaded from NCBI (SRR1029852.sra) and assembled with SPAdes [37]. The annotation was completed using Prokka [38]. Selection of Ehrlichia sequence used in the probe was carefully chosen to avoid hybridization with the *I*. *scapularis* endosymbiont genome (accession # JFKF01000080; locus tag REISMN_04040). Amplifying branched DNA was hybridized to the probe to increase signal strength, followed by hybridization with a label probe that utilized Fast Red as substrate (2-amino-5-chlorotoluenehydrochloride tablets dissolved in napthol) to provide both red chromogenic and red fluorescent labelling.

### Imaging

Sections affixed to slides were viewed on an Olympus DSU (disc scanning unit) confocal microscope equipped with a Q-Fire digital camera, as well as DAPI and mCherry filters and a Photometrix QuantEm 512 SC camera for fluorescent microscopy image capture. MetaMorph Premier (Molecular Devices, Sunnyvale, CA, USA) imaging software in conjunction with ImageJ (U.S. NIH, Bethesda, MD, USA) and Adobe Photoshop software were used to acquire and process images. The *Smart Sharpen filter* and *Brighten*/*Contrast* tools in Photoshop CS4 were used to enhance contrast in TEM images and to correct brightness levels in brightfield images.

### Transmission Electron Microscopy

Freshly molted adults infected with EmCRT as larvae were bisected anterior to posterior using a sterile # 15 surgical blade (Southmedic, Barrie, ON, Canada). These specimens were immediately fixed using a modified version of Ito’s fixative [39,40], first under vacuum for 10 min, then submerged at 4°C overnight. Following three 5 min rinses with 0.1 M sodium cacodylate buffer, 1% osmium tetroxide was used for secondary fixation (3.5 h at room temperature) prior to a second wash in 0.1 M sodium cacodylate buffer. Rinsed fixed specimens were stained with 0.5% uranyl acetate overnight at 4°C, and dehydrated in an ascending series of ethanol (20% for 10 min, 40% for 10 min, 60% for 10min, 80% for 10min, 95% and 100% for 10 min x 3), followed by an acetone to resin transition where >99% acetone was used for a total of two 10 min washes. Samples were then immersed in resin diluted 1:1 by volume with acetone for 30 min using a rotator and subsequently removed from the mixture and infiltrated with undiluted Spurr Low Viscosity Resin (Sigma-Aldrich, St. Louis, MO, USA) for 24 h. Resin containing ticks was poured into blocks and polymerized at 70°C for 24 hours. Ultrathin sections were cut using a diamond knife on an Leica Ultracut LCT ultramicrotome (Leica Microsystems), collected on formvar/carbon-coated copper grids, and stained at room temperature for 20 min in 3% aqueous uranyl acetate followed by 3 min in Sato's lead stain. Sections were visualized and imaged with a Philips CM12 model transmission electron microscope (FEI, Hillsboro, OR, USA) operating at 60 kV at the University of Minnesota Imaging Centers-St. Paul. Anatomical structures were identified with the aid of several literature sources [41–43].

## Results

### In situ hybridization (ISH)

**Fig 1:**
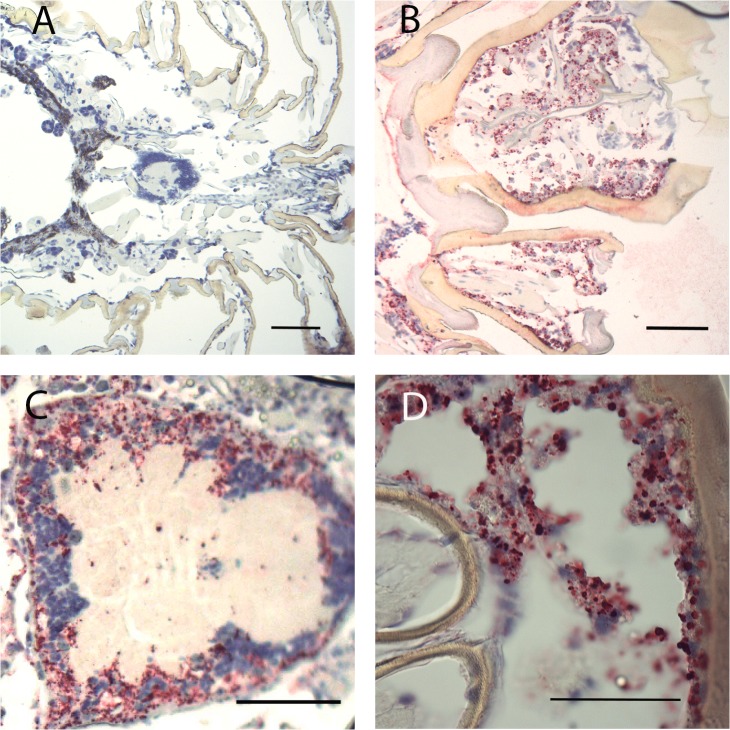
Whole tick sections subjected to ISH assay. (A) Nymph with non-apparent EML infection. (B) Female basis capitulum showing heavy infection of epithelial tissues. (C) Male synganglion showing heavy infection of outer cortex. (D) Transversal section through male basis capitulum with cheliceral sheathes shown at left. Red indicates bacterial probe hybridization, blue indicates nuclear staining. Scale bars (SB) A, B, C = 10μm, D = 5 μm.

**Fig 2:**
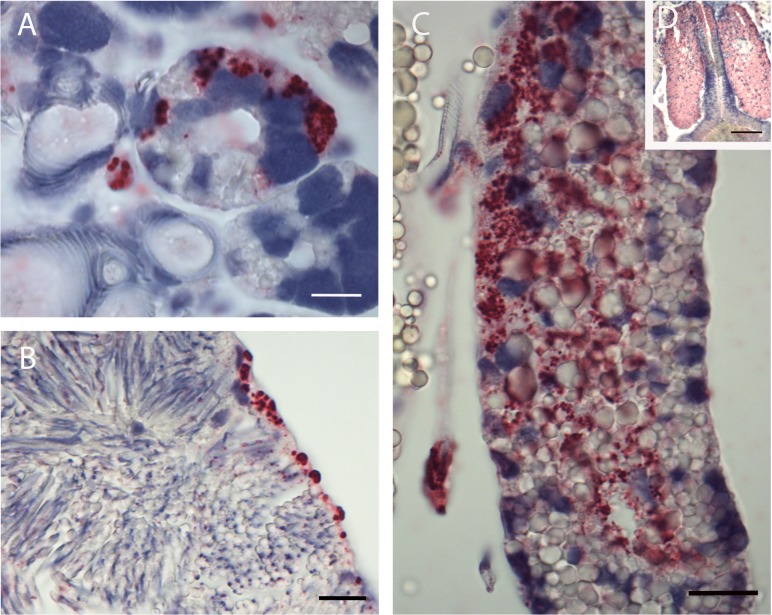
Whole tick sections subjected to ISH assay. (A) Acini and salivary ducts of infected adult female SB = 1μm. (B) Spermatozoa of adult male with infected lining sheath. SB = 1μm. (C) Infected adult male accessory gland (single lobe). SB = 2μm. (D) Infected adult male accessory gland (multiple lobes). SB = 10μm.

**Fig 3:**
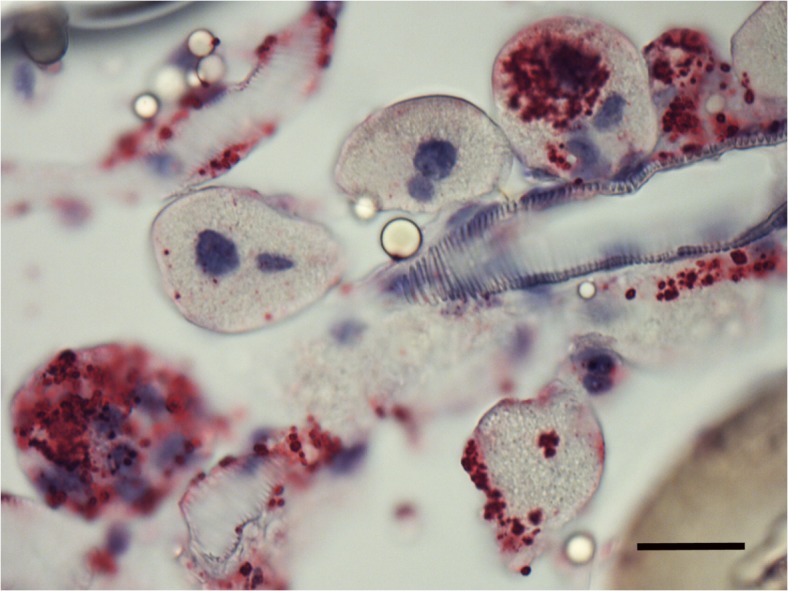
Whole tick sections subjected to ISH assay. Salivary gland of adult male showing multiple infected acini and salivary ducts. SB = 2μm.

**Fig 4:**
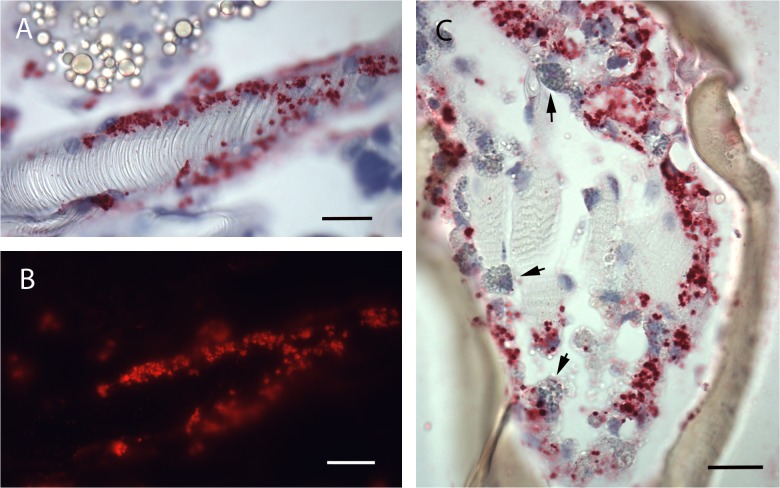
Whole tick sections subjected to ISH assay. (A) Infected epithelial cells lining tracheole in adult male (bright field microscopy). (B) Same specimen as (A) imaged using fluorescent red filter. (C) Appendage of infected adult male. Arrows indicate hemocytes. SB = 2μm.

Microscopic examination of sectioned *I*. *scapularis* that had undergone ISH demonstrated great variability in bacterial load between individual ticks. While many ticks showed broadly disseminated infections that were easily visible by bright field microscopy facilitated by the chromogenic label, in others, infection load was light enough to require the use of fluorescent microscopy to detect probe signal (Fig. 1A, bright-field). Light infection loads were those in which few small morulae were scattered throughout tissues or restricted to one or two organs, and no obvious pathogenic changes were observed. Heavy infection loads (Fig. 1B) were defined as those that affected the entire tick, including leg muscle, synganglion, salivary glands and male accessory glands, and induced significant and wide-spread tissue damage. Because of the predilection of *E*. *muris* for the male accessory glands, males were always considered heavily infected. Females and nymphs observed in this study displayed similar infection patterns, with several individuals of each group showing widespread infection throughout their anatomy. While ehrlichiae were found in tissues throughout the tick, they appeared to preferentially invade epithelial cells. Salivary glands (Fig. 2A & Fig. 3), outer cortex regions of the synganglion (central nervous system) (Fig. 1C) and epithelial cells surrounding the tracheal complex (Fig. 4A & 4B) were the most frequent sites of infection among the ticks sampled. Interestingly, some salivary gland acini harbored large numbers of ehrlichiae, while neighboring clusters were lightly or uninfected, suggesting a possible tropism for specific types of acini that was further supported by TEM findings in this study [15,43]. Similarly, we consistently observed ehrlichiae in cells forming the peripheral cortical region of the synganglion while the internal neuropile was largely free of morulae or contained a few scattered groups of bacteria (Fig. 1C). Ovarian tissues showed no evidence of active infection, an unsurprising observation given that transovarial transmission has not been observed within the genus *Ehrlichia* [44].

Conversely, male reproductive tissues showed varying susceptibilities, as the accessory glands of several individuals contained numerous EML bacteria. Ehrlichiae were especially apparent in the secretory cells of the latero-dorsal lobes (Fig. 2C), while adjacent lobes of the accessory gland and seminal vesicle appeared to be much less susceptible (Fig. 2D). Spermatids and spermatocysts did not show evidence of intracellular bacteria, and testes appeared relatively free of EML, with probe hybridizing only to cells forming the capsule (Fig. 2B). Similar to females and nymphs, salivary glands, synganglion, and epithelial cells in males were heavily infected.

In heavily infected individuals, EML invaded the connective tissue and muscle throughout the legs, appearing to spread along the epithelial cells lining tracheoles extending throughout the body (Fig. 4). While we did observe EML RNA within hemocytes, no morulae were evident (Fig. 4C), leaving it unclear whether the cells played a role in dispersion of EML, as reported for *A*. *phagocytophilum* [26], or whether we simply detected an immune response and bacterial phagocytosis. Many of the infected tissues were adjacent to interstitial compartments, which suggested possible cell-free dispersion of EML. Of additional interest, we noted a visually significant load of bacteria in the basis capitulum and mouthparts of several individuals. This could possibly reflect route of initial bacterial exposure during acquisition feeding as well as potentially represent a factor in tick to host transmission (Fig. 1B & 1D). In contrast to previous publications localizing *Anaplasma* spp., infection of gut tissues was infrequent and light.

In several individuals, tissues containing large numbers of ehrlichiae showed signs of pathogenic changes, particularly within the appendages where muscle tissues appeared to be deteriorating or detached from connective tissues and the chitinous exoskeleton. Additional areas of apparent tissue damage included frayed lining of the synganglion, and lesions within synganglial tissue and the lumen of male accessory glands (Fig. 2C).

### TEM

**Fig 5:**
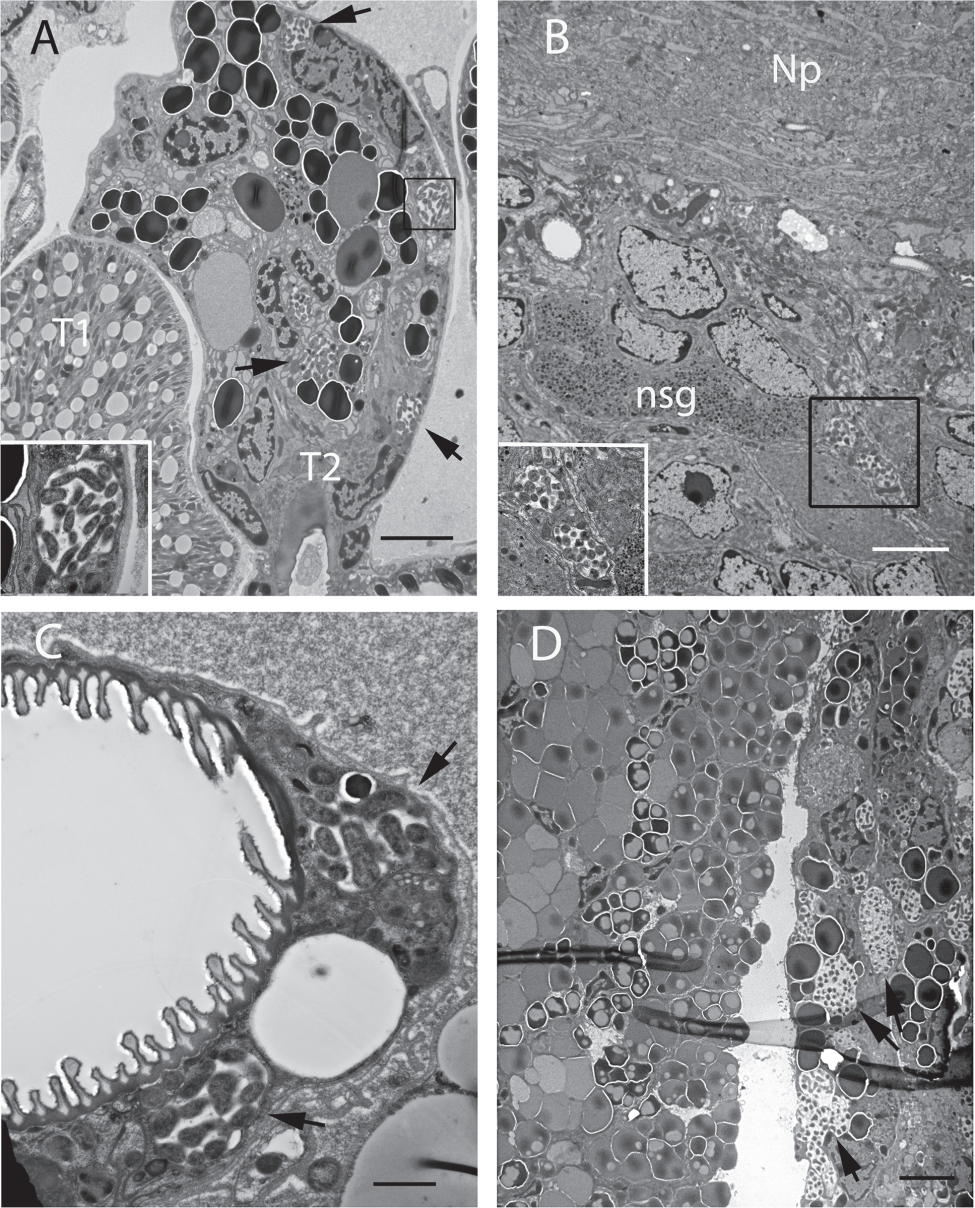
TEM of whole tick sections. (A) Male salivary gland showing Type I and Type II acini. Arrows indicate EML morulae. Inset shows morula at higher magnification. (B) Synganglion of infected adult male tick. Np = neuropile, nsg = neurosecretory granules. Inset shows morulae at higher magnification. (C) Infected epithelial sheath surrounding a tracheole. (D) Adult male accessory gland with heavy infection of lobe on the right. SB A,B,D = 5μm, C = 1μm.

Transmission electron microscopy was used to confirm results of the ISH assay. Ehrlichial bodies displaying an outer cell wall membrane and an inner cytoplasmic membrane were identified within morulae in both male and female *I*. *scapularis* (Fig. 5A & 5B insets). While variable with respect to cell type, infected tick cells typically exhibited 1–3 morulae per cell, with 10 to 20 bacteria per section, in contrast to light microscopic observations of EML-infected tick cell cultures where cells may contain as many as six morulae that may harbor dozens of bacteria [11]. Morulae in most tissues, with the exception of the male accessory glands were smaller and contained fewer bacteria than described for *A*. *marginale* and *E*. *muris* [21,30]. EML bacteria were observed in multiple organs and tissues including salivary gland (type II and type II acini) (Fig. 5A), synganglion (Fig. 5B), the tracheal complex (Fig. 5C), male accessory gland and connective tissues (Fig. 5D). Ehrlichiae were not observed in type I (non granulated) acini. Both reticulate and dense core organisms were present.

## Discussion

An organism’s capacity to utilize the contrasting environments offered by arthropod and mammalian host cells depends upon a diverse set of physiologic abilities. Differences in transcription, antigenicity, developmental rate and appearance have been observed between *Anaplasmataceae* cultured in tick and mammalian cell lines as well as *in vivo* infection of different hosts and tissues, reflecting a potentially unique set of interactions between the bacteria and a particular environment [15,21,24,45–52]. These comparisons are vital for understanding the functions of the genes that allow intracellular pathogens to navigate highly disparate host systems and cell types using a limited genome. Accordingly, results of this study address a key deficit in existing knowledge of the arthropod phase of the life cycle of ehrlichiae and provide a basis to compare and contrast with the mammalian stage of infection. In addition to improving our understanding of a potentially important model organism for human ehrlichiosis [19], our findings may contribute toward the study of other human pathogens vectored by *I*. *scapularis*, including *A*. *phagocytophilum*.

The application of ISH to whole sectioned ticks enabled a sensitive and specific means to screen entire ticks for EML infection rather than limiting our search to specific tissues. EML were identified in tissues beyond the expected targets, i.e., midgut and salivary glands, and the assay allowed us to pinpoint the location of bacteria to a specific region of an organ while retaining exact anatomical context. Electron micrographs supplemented the ISH images, displaying EML tropism for neural cells in the outer cortex region of the synganglion, particularly along the border of the neuropile. Salivary gland images facilitated the identification of specific acinus types and confirmed the heavy and regionally specific foci of infection in the male accessory gland, another secretory organ. Broad areas of general probe hybridization (pink regions in Fig. 2D) in these structures suggest that ehrlichial RNA is present within the secretory fluids, possibly as a result of degraded bacteria released from heavily infected lysing cells. At present, published work on the anatomy and function of this organ is limited, especially for *Ixodes* spp. Further study in this area could provide a clearer picture of why certain lobes are associated with heavy infection while proximal regions of the gland are uninfected, and if there is an effect on male reproductive fitness. Cytopathic effects were also apparent in muscle and connective tissues within appendages such as the legs and palps, and throughout the basis capitulum. Although these regions are proximal to the alimentary canal, it is not known whether bacteria residing there can access salivary ducts or esophagus during transmission feeding, unless severe damage from heavy infections would sufficiently compromise tissue integrity to allow leakage into the food canal. In contrast to previous reports for *E*. *muris* infection of *Ixodes persulcatus* [30], salivary gland cells appeared largely intact.

Somewhat unexpectedly, we found that ehrlichiae were highly disseminated throughout various tick tissues, with a strong affinity for epithelial cells. The contrast observed between several heavily infected nymphs and several lightly infected females suggested that the degree of infection (EML load) was not necessarily dependent on developmental stage, and may be a result of individual tick susceptibility or bacterial dosage related to feeding position on the host. Irrespectively, males consistently displayed heavy infections. Salivary glands were heavily colonized in all life stages where infection was chromogenically visible while the gametes of both sexes were free of bacterial colonies, indicating enzootic horizontal transmission as the probable route of pathogen maintenance in nature.

Visual recognition of salivary glands, synganglia, skeletal muscle, and epithelial tissues as sites harboring EML bacteria coincides with prior reports localizing *Anaplasma* and *Ehrlichia* spp.[15,20–23,26,27,30]. However, the ehrlichial load in some ticks in our study was more extensive than what was previously assumed for *Anaplasmataceae* other than *A*. *marginale*. Because we infected ticks as larvae, it is possible that the extended period of infection or the additional physiological tissue reorganization during a second molt (nymph to adult) could have led to more disseminated ehrlichial colonization of adult ticks than in adults infected as nymphs. Yet we also observed several nymphs with heavy bacterial burdens, suggesting other factors such as host bacteremia level at the time of feeding, position of tick during feeding, or reduced tick immune responses to EML as possible explanations. Despite heavy colonization of essential tissues such as synganglia, which frequently appeared to show pathologic changes, we did not note any adverse reactions to EML in ticks such as difficulty feeding or reduced survival under laboratory conditions. Yet it is possible that under more stressful conditions in the wild, EML may have a discernable negative impact on vectors and possibly account for the low prevalence of these infections in wild-caught ticks [11,13]. A broad distribution throughout the vector has previously been considered to be indicative of a pathogenic species rather than a symbiont in other Rickettsiales [53–55] typically found at low frequencies in wild tick populations. Determining the potential impacts of advanced EML infection on the fitness of ticks, including their ability to access hosts and overwinter survival might contribute toward a clearer picture of the epidemiology of this organism.

Future research efforts should describe the pathogen-vector relationship sequentially through the processes of acquisition, internal development, dissemination, and transmission to host. Doing so may uncover key points for disruption of the transmission process through vaccines or therapeutic approaches.
